# The relationship between psychosocial factors and reported disability: the role of pain self-efficacy

**DOI:** 10.1186/s12891-021-04955-6

**Published:** 2022-01-03

**Authors:** Antonio J. Varela, Kathryn W. Van Asselt

**Affiliations:** 1 School of Physical Therapy, Arkansas Colleges of Health Education, Fort Smith, USA; 2 Psychology Department, Capella University, Minneapolis, USA

**Keywords:** Chronic pain, Low back pain, Biopsychosocial, Depression, Fear, Catastrophizing, Self-efficacy, Reported disability

## Abstract

**Background:**

Chronic pain and the accompanying level of disability is a healthcare crisis that reaches epidemic proportions and is now considered a world level crisis. Chronic non-specific low back pain (CNLBP) contributes a significant proportion to the chronic pain population. CNLBP occurs with overlapping psychosocial factors. This study was design to investigate specific psychosocial factors and their influence on reported disability in a population with CNLBP.

**Methods:**

The specific psychosocial factors examined included fear, catastrophizing, depression, and pain self-efficacy. This cross-sectional correlational study investigated the mediating role between pain self-efficacy, the specific psychosocial factors, and reported disability. The study recruited 61 female and 29 male participants from physical therapy clinics. The participants were between 20-to-60 years of age and diagnosed with CNLBP. All participants completed the Fear Avoidance Belief Questionnaire, The Pain Catastrophizing Scale, The Patient Health Questionnaire-9, The Pain Self-Efficacy Questionnaire, and The Lumbar Oswestry Disability Index. The battery of questionnaires measured fear of physical activity, pain catastrophizing, depression, pain self-efficacy, and reported disability. Multivariate regression and mediation analyses was used to analyse the data.

**Results:**

The principal finding was a strong inverse relationship between pain self-efficacy and reported disability with a *p*-value < 0.001. Further, pain self-efficacy was considered a statistical mediator with consistent *p*-value < 0.001 for the specific psychosocial factors investigated within this data set. Pain self-efficacy was considered to have a mediating role between reported fear of physical activity and disability, reported pain catastrophizing and disability, and reported depression and disability. Additionally, age and reported pain levels proved to be statistically significant. Adjustments for age and pain level did not alter the role of pain self-efficacy.

**Conclusion:**

The results identified a mediating role for pain self-efficacy between the specific psychosocial factors (fear, catastrophizing, and depression) and reported disability. Pain self-efficacy plays a more significant role in the relationships between these specific psychosocial factors and reported disability with CNLBP than previously considered.

## Background

Chronic pain and reported disability have reached epidemic proportions [1, 2]. This epidemic affects an estimated 37–41% of people worldwide and nearly 20% of the United States population [3–5]. Chronic pain ranks first as the cause of the most years lived with disability [6–9]. Individuals experiencing low back pain (LBP) delineate the greatest percentage of people with chronic pain [10]. Chronic low back pain (CLBP), the most common musculoskeletal condition, accounts for approximately half of the United States chronic pain population and has an annual incidence rate that accounts for more than 1/3 of the global health conditions [3, 7, 11–15].

Distinct musculoskeletal causes for chronic pain and reported disability are found in 10–20% of the chronic pain population [10, 16, 17]. Chronic non-specific low back pain (CNLBP) exemplifies those with chronic pain [18]. CNLBP is a major contributor to the overall chronic pain epidemic with its high prevalence and recurrence rates [7, 10, 19–21]. The prevalence and recurrence rates may be associated with passive procedures and interventions utilized within chronic pain management practices [22–24]. Additional studies conclude that psychosocial factors are associated with baseline and recurring clinical characteristics in patients with chronic pain [16, 25]. A broad range of cognitive and affective psychosocial factors expose vulnerabilities and undermine an individual’s ability to manage pain [26]. The lack of specific treatable pathology, the passivity of current healthcare models, and dismal outcomes underscore the psychosocial factors as appreciable contributors to the development and perpetuation of chronic pain and disability. The momentum of research and interventions for chronic pain include evaluations and treatments predicated on psychosocial factors [27, 28]. However, the implementation of that research falls short of sustainable outcomes [23].

Many chronic pain cases move through healthcare in fragmented stages without comprehensive reviews of the clinical interventions, psychosocial factors, compliance consistency, and related barriers to recovery [29, 30]. These healthcare practices are enabled by a patient’s lack of problem-solving skills, coping skills, and self-confidence to independently manage pain and disability [31–33]. These attributes are associated with decreased self-efficacy beliefs [34]. Self-efficacy refers to the belief in one’s capabilities and the confidence in one’s abilities to organize, perform, and complete the courses of action required to achieve a particular behaviour or outcome [31]. Self-efficacy in the context of pain may exist as a common denominator between other psychosocial factors [35]. Pain self-efficacy includes beliefs about an individual’s ability to control pain, regulate associated emotions, maintain work activities, communicate to healthcare providers, and appropriately utilize pain management strategies [36]. Pain self-efficacy as a potential mediator of reported disability influences associated psychosocial factors. However, the chronic pain literature lacks investigations of the relationships between pain self-efficacy, psychosocial factors, and reported disability. This research explores the relationship between specific psychosocial factors, pain self-efficacy, and reported disability in the biopsychosocial and social cognitive contexts of chronic pain.

Recent literature asserts self-efficacy has the strongest magnitude for mediating indirect effects of most CLBP treatments and outcomes [37]. Investigating pain self-efficacy’s influence on reported disability may provide critical insight into chronic pain management. Self-efficacy may be a determinant of reported disability when the inverse relationship between them is highlighted [37, 38]. This research begins to address the need to shift toward positive psychology and potential adaptive behaviours that build on the current understanding of maladaptive responses. This manuscript investigates pain self-efficacy’s role in the relationships between specific psychosocial factors and reported disability in those with CNLBP. This research may help clarify the role of pain self-efficacy in chronic pain.

## Methods

Four research questions were formulated to examine the relationship between specific psychosocial factors and reported disability. The research questions are listed below.

- *Research Question 1 (RQ1):* Is there a correlational relationship between pain self-efficacy and reported disability?
- *Research Question 2 (RQ2):* Does pain self-efficacy serve as a mediating variable between fear of physical activity and reported disability?
- *Research Question 3 (RQ3):* Does pain self-efficacy serve as a mediating variable between pain catastrophizing and reported disability?
- *Research Question 4 (RQ4):* Does pain self-efficacy serve as a mediating variable between depression and reported disability?

### Research design

The study was a non-experimental quantitative cross-sectional correlational design investigating the relationship between specific psychosocial factors, pain self-efficacy, and reported disability. The study specifically investigated how pain self-efficacy changed the relationships between fear, catastrophizing, depression, and reported disability. This correlational study was designed on multivariate regression and mediation analyses. Mediation analysis offered a method of exploring relationships between predictors, outcomes, and intervening variables [39].

### Setting and participants

A purposeful sample was drawn from four different physical therapy clinics within four cities in the continental United States. The participants were adults ages 20 to 60 diagnosed with an exacerbation of CNLBP. This diagnosis was also acceptable as non-specific low back pain (LBP), LBP of non-specific origin, back pain without radiculopathy, condition of the back and spine without surgical indication, and LBP site unspecified. Primary care practitioners referred the participant sample to physical therapy. All participants met the inclusion criteria and signed a written informed consent. Data for each participant were collected at the intake of a physical therapy episode. Responses to standardized questionnaires were obtained through paper format. The data collected were based on questionnaires designed to capture the specific psychosocial measures and levels of reported disability.

The outcome variable in this study was reported disability. The predictor variables were reported pain self-efficacy, fear of physical activity, pain catastrophizing, and depression. All data were collected by the principal investigator with assistance from co-investigators. Subjects from 20-to-60 years of age diagnosed with CNLBP were invited to participate. The exclusion criteria were anyone with a work-related injury, litigation for compensation from an injury, history of cancer, and specific lumbar spine nerve related involvement such as a loss of reflexes.

### Power analysis

An a priori power analysis was conducted to assess the study sample’s ability to maximize the probability of accepting a true effect in a population. The power analysis was conducted using G*Power 3.1.9.2 [40], which determined a sample size of 89 participants would be needed. The power calculation was based on a concentrated power of 80% with ES = 0.15, Alpha = 0.05 and using the 5 identified predictors [40].

### Instruments

Study participants completed a standard medical intake questionnaire. The participants also completed a battery of six questionnaires related to CLBP including the Pain Self-Efficacy Questionnaire [41, 42] and the Lumbar Oswestry Disability Index [43]. The psychosocial variables of fear, catastrophizing, and depression were assessed with the Fear Avoidance Behaviour Questionnaire [44], the Pain Catastrophizing Scale [45], and the Patient Health Questionnaire-9 [46]. The data gathered was considered continuous data. The measurement tools were considered restrictive as they require the participant to commit to a continuous scale. All the questionnaires have been validated in chronic musculoskeletal and chronic pain disorders including LBP [36, 43, 44, 46–49].

The Pain Self-Efficacy Questionnaire (PSEQ) investigated pain self-efficacy. The constructs measured included knowledge and skills to confidently motivate and maintain one’s abilities for everyday activities. The PSEQ also investigated the cognitive resources required to perform and successfully complete a specific task within the context of pain [36, 42]. The psychometric properties of the PSEQ demonstrated reliability, validity, and internal consistency with a Cronbach Alpha score of 0.92 [41].

The Lumbar Oswestry Disability Index [50] (ODI) investigated reported disability associated with all stages of low back pain. The questionnaire provided a percentage score of one’s perceived disability [43]. No specific constructs or content validity related to the lumbar ODI was found in the literature. The properties of the lumbar ODI demonstrated reliability, validity, and internal consistency with a Cronbach Alpha score of 0.71 to 0.871 [43].

The Fear Avoidance Behavior Questionnaire (FABQ) investigated pain related fear beliefs about general physical and work-related activity and identifies those at risk of prolonged disability. The construct of the FABQ investigated fear of movement or re-injury [44]. The focus of the FABQ was on pain-related beliefs and expectations rather than the critical attributes of phobias [51]. The internal consistency of the FABQ had a Cronbach’s alpha estimate ranging from 0.70 to 0.83 [44]. This research focused on fear of physical activity, the FABQpa subscale. The FABQpa calculations did not include the distractor question. The work-related scale (FABQw) was not used due to the exclusion criteria. The test-retest reliability for the FABQpa presented with a Pearson *R* = 0.84 to 0.88 [52, 53]. FABQpa scores were identified as a predictor of disability and are significantly correlated with both pain intensity and disability in patients with LBP [47, 53–55].

The Pain Catastrophizing Scale (PCS) assessed the degree of catastrophic thinking associated with LBP. Research beyond the original work supports a multidimensional conceptualization of catastrophizing that included rumination, magnification, and helplessness [45, 56, 57]. Test-retest reliability was good and was reported with Pearson R scores equal to 0.70 to 0.77. Cronbach’s alpha estimates for the PCS range from 0.85 to 0.92, which demonstrated internal consistency [45].

Depression was assessed by the Patient Health Questionnaire - 9 (PHQ-9). The PHQ-9 constructs represented depression, facilitated the severity of depression, and correlated with increasing disability [46, 58]. The PHQ-9 had internal consistency established with a Cronbach’s alpha estimate of 0.87 and 0.85 [46, 59]. The test-retest reliability of the PHQ-9 was also excellent [46]. The PHQ-9 provides a criterion-based diagnosis of depression with comparable sensitivity (0.83) and specificity (0.72), and strong correlations with well-established measures consistent with depression and double its length [59].

### Data analysis

The study utilized Jamovi, a graphical user interface built on top of the R statistical environment. Jamovi is a free open-source statistical software (https://www.jamovi.org/about.html). Statistical significance was established a *p* = 0.05. All the data from the questionnaires, for the predictor and outcome variables, were continuous data allowing for correlational analysis. Within the correlational design, the statistical techniques of multivariate regression, mediation and moderation analysis were used to investigate the relationships between pain self-efficacy, fear of physical activity, pain catastrophizing, depression and reported disability. The study measured the relative strength of the linear relationship between pain self-efficacy, fear, catastrophizing, depression, and reported disability. The data were analysed consistent with hypotheses testing to see if pain self-efficacy mediated the relationship between the other psychosocial factors and reported disability.

Study participants completed a standard medical intake questionnaire consisting of questions related to age, gender, and pain level. This information was then collated into a framed format. Columns for each of the questionnaire scores, columns for the individual questions, and columns for specific demographic and pain data were included within the framed format. The rows designated participants with their coded demographic information. The questionnaires were scored according to the instrument guidelines. The results were imported into Jamovi statistical software.

The questionnaires were calculated separately per guidelines. Continuous variables were assessed for normal distributions and extreme outliers. A multiple linear regression analysis was conducted on these variables for the investigation. The regression analysis and multiple regressions statistics were used to complete data analysis.

## Results

The sample consisted of 90 participants who met the inclusion criteria, 68% (*n* = 61) were females and 32% (*n* = 29) males. The demographics collected only include gender, age, and pain level. The mean age of participants was 44.4 and the mean pain level was 5.53. Participant demographic are provided in Table 1. Two participants in the study were excluded after completion of the questionnaires. One participant had a spinal fracture and the other participant decided to litigate. No participants decided to withdraw from the study. The data included summative scores representing each of the questionnaires and their respective psychosocial factors. The statistics were slightly skewed by the presence of more females; however, this skewness did not affect the *p*-values on any hypothesis tests within the regression analysis.

**Table 1:** Descriptive Statistics Demographics

	Population	Age	Pain Level
N	90 29 male (32.2%) 61 female (68.8%)	****	****
Mean	******	44.4	5.53
Median	******	47.0	6.00
SD	0.470	11.5	2.09
Minimum	0 – female^a^	20.0	1.00
Maximum	1 – male^a^	60.0	9.00

^a^0/1 female / male coding scheme for regression analysis

### Descriptive statistics and data screening

#### Research question 1(RQ1)

**Fig. 1:**
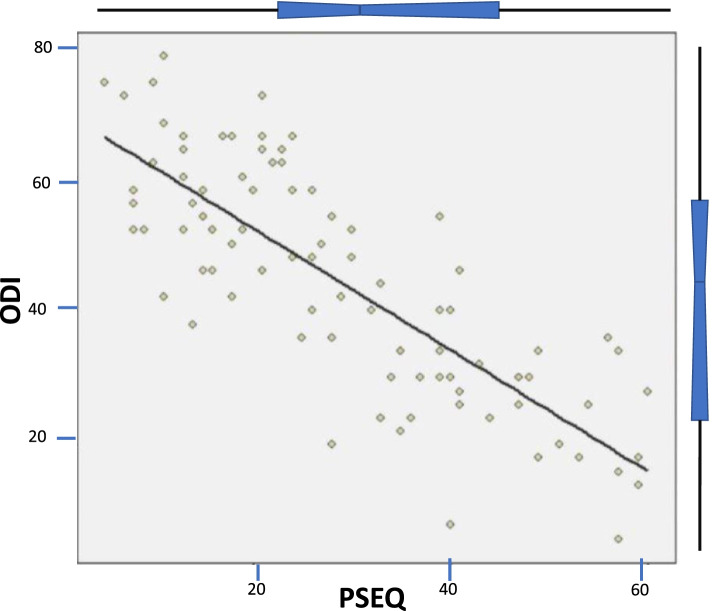
Bi-variate scatterplot of PSEQ and ODI

Analyses began with the bi-variate scatterplot, Fig. 1, to assess the relationship between the PSEQ and the ODI. The bi-variate scatter plot reflected a strong, negative association between the results of the PSEQ and ODI. The estimated Pearson correlation coefficient was − 0.806 with a *p*-value < 0.001, indicating a negatively statistically significant association between PSEQ and ODI. There was sufficient evidence to support the hypothesis. However, significant correlations of pain level and age were unexpectedly discovered. Therefore, the full description of the relationship between PSEQ and ODI required adjustment for pain level and age.

A simple linear regression of PSEQ onto ODI was completed. A Pearson correlation coefficient of − 0.806 with an interpretation of the R^2^, as an effect size measure, suggested that 65% of the variability within the ODI was explained by applying the PSEQ score. Initially, the PSEQ parameter had an estimate of − 0.907, indicating that a unit increase in PSEQ was met with an expected (or average) 0.907-point decrease in the ODI. However, this changed with the statistical adjustments for pain level and age. The negative correlation between PSEQ and ODI remained significant (*p* < 0.001) after adjusting for reported pain level and age. The impact for a one unit increase in reported PSEQ was an expected − 0.670-unit decrease in the ODI. Every single positive point change in pain self-efficacy was seen with two-thirds of a point decrease in reported disability. Post hoc assessment of residual and Q-Q plots revealed there were no outliers and residuals, which suggests that *p*-values were unbiased.

#### Research question 2(RQ2)

The assessment of the relationships in RQ-2 required multiple regression equations. The covariate adjustments of pain level and age remained consistent through remaining research questions. The model of dependence to answer RQ2 focused on the physical activity portion of the FABQpa. The initial step required relationship analysis between FABQpa and ODI. The absence of a correlating relationship between FABQpa and ODI was required to establish that pain self-efficacy had a mediating effect in the relationship between FABQpa and ODI. The first model required the ODI being predicted by a multiple linear regression of FABQpa, pain level, and age as seen in Table 2.

**Table 2:** ODI, Pain, Age, and FABQpa Model Coefficients

95% Confidence Interval							
Predictor	Estimate	***SE***	Lower	Upper	***t***	***p***	Stand. Estimate
Intercept	−4.805	6.811	−18.3441	8.734	−0.706	0.482	0.646
Pain Level	5.243	0.657	3.9366	6.550	7.977	<.001	
Age	0.254	0.110	0.0350	0.473	2.306	0.024	0.172
FABQpa	0.527	0.247	0.0356	1.019	2.132	0.036	0.171

The ODI acted as the Intercept in Table 2. The interpretation required that both pain level and age remained statistically significant (< 0.001and 0.024, respectively). Additionally, the *p*-value on the FABQpa coefficient was 0.036 (95% confidence interval), which provided sufficient evidence to reject the null hypothesis. A relationship between FABQpa and ODI could not be established when adjusting for pain level and age. This enabled the non-direct association between FABQpa and ODI facilitating mediation analysis of PSEQ. Prior to this assessment, the post hoc consideration of variance inflation factors revealed there were no correlations between factors affecting the *p*-values (or estimates of the coefficients). Further, a post hoc assessment of residuals via Q-Q plot analysis was completed in terms of goodness of fit. There was no evidence of lack of fit in this model. The results could not identify a reason to suggest that this model was inappropriate.

The analysis in Table 3 demonstrated a direct relationship between FABQpa and PSEQ. PSEQ acted as the Intercept in Table 3. The *p*-value of 0.029 of FABQpa provided sufficient statistical evidence when predicting the role of PSEQ. In this case, the statistics provided significant evidence to further reject the null hypothesis. This regression analysis determined how pain self-efficacy acted on fear of physical activity. Pain self-efficacy changed the structure of the relationship between FABQpa and ODI. The variance inflation factors presented no evidence of issues with the results. Likewise, the Q-Q plot of residuals did not identify any problem with the model fit. The final step was assessing if PSEQ mediated the relationship between FABQpa and ODI and to fit the multiple linear regression with all terms. The ODI acted as the intercept in this model seen in Table 4. There was a statistically significant (*p* < 0.001) association between the PSEQ and the ODI. Full mediation was present when the significance of the *p*-value on PSEQ rendered the *p*-value on the FABQpa as no longer significant.

**Table 3:** PSEQ, Pain, Age, and FABQpa Model Coefficients

95% Confidence Interval							
Predictor	Estimate	***SE***	Lower	Upper	***t***	***p***	Stand. Estimate
Intercept	64.369	6.829	50.793	77.9453	9.43	<.001	−0.5338
Pain Level	−3.846	0.659	−5.156	−2.5354	−5.83	<.001	
Age	−0.113	0.110	−0.332	0.1063	−1.02	0.308	−0.0865
FABQpa	0.550	0.248	−1.043	0.0575	−2.22	0.029	−0.2010

**Table 4:** ODI, Pain, Age, FABQpa, and PSEQ Model Coefficients

95% Confidence Interval							
Predictor	Estimate	***SE***	Lower	Upper	***t***	***p***	Stand. Estimate
Intercept	37.252	7.3796	25.5796	51.925	5.048	<.001	0.3367
Pain Level	2.731	0.5902	1.5572	3.904	4.627	<.001	
Age	0.180	0.0841	0.0126	0.347	.138	0.035	0.1222
FABQpa	0.168	0.1932	−0.2166	0.552	0.867	0.388	0.0544
PSEQ	−0.653	0.0817	−0.8159	−0.491	−7.995	<.001	−0.5804

The second research question determined that pain self-efficacy mediated the relationship between fear of physical activity and reported disability when adjusting for reported pain level and age. Research questions 3 and 4 followed the same methodology.

#### Research question 3(RQ3) and 4(RQ4)

The model of dependence to answer RQ3 and RQ4 focused on PCS and PHQ-9, respectively. Neither the PCS and ODI or PHQ-9 and ODI could not have a correlating relationship to examine the mediating effect of PSEQ. For RQ3, the first model required the ODI to predict PCS, pain level, and age. This was a similar requirement of RQ4 and PHQ-9.

The ODI acted as the intercept for both. The *p*-values of both pain levels and age remained statistically significant. Additionally, the *p*-value for PCS was 0.005 while the *p*-value for ODI was 0.714. Likewise, the *p*-value for PHQ-9 was 0.001 and the *p*-value for ODI was 0.701. A relationship either between PCS and ODI or PHQ-9 and ODI was not identified. The statistical evidence was sufficient to reject both null hypotheses.

Post hoc assessment of residuals and variance inflation factors yielded no evidence of a poorly fitted model for the relationship between PCS and ODI for RQ3. However, a significant standard residual of 2.8 was discovered within the relationship between PHQ-9 and ODI in RQ4. This residual did not demonstrate any leverage. No concerns with the appropriateness or variance inflation factors developed over the effect on the analysis. The Q-Q Plot also demonstrated minor evidence of non-normality, but not significant enough to modify the interpretations.

The mediation analysis continued to assess the role pain self-efficacy played in the relationship between catastrophizing and reported disability for RQ3. Mediation analysis continued to assess the role of pain self-efficacy in the relationship between depression and reported disability for RQ4. This began with the regression of the potential mediator PSEQ on ODI as previously established in RQ1. The investigation into RQ3 continued by regressing PCS on PSEQ and finally regressing PSEQ and PCS on ODI to assess overall mediation. The investigation into RQ4 regressed PHQ-9 on PSEQ and concluded by regressing PSEQ and PHQ-9 on ODI to assess overall mediation.

PSEQ acted as the Intercept for RQ3. The *p*-value of < 0.001 on the PCS indicated that PCS was a statistically significant predictor of PSEQ after adjusting for pain level and age. The final step was assessing if PSEQ mediated the relationship between PCS and ODI and to fit the multiple linear regression with all terms. Full mediation was established when the *p*-value on the PCS was no longer significant in the presence of a significant PSEQ *p*-value. The ODI acted as the Intercept in Table 5. This model completed the mediation analysis of PSEQ within the relationship between PCS and ODI. The estimate of PCS was no longer statistically significant as noted by *p* = 0.768. The indication was that the relationship between pain catastrophizing and reported disability changed when pain self-efficacy was introduced into the regression model.

**Table 5:** ODI, Pain, Age, PSC and PSEQ Model Coefficients

95% Confidence Interval							
Predictor	Estimate	***SE***	Lower	Upper	***t***	***p***	Stand. Estimate
Intercept	38.9470	7.3639	24.3055	53.588	5.289	<.001	0.3434
Pain Level	2.7851	0.6021	1.5879	3.982	4.625	<.001	
Age	0.1868	0.0852	0.0174	0.356	2.192	0.031	0.1269
PCS	0.0262	0.0884	−0.1495	0.202	0.296	0.768	0.0217
PSEQ	−0.6594	0.0873	−0.8329	−0.486	−7.556	<.001	−0.5858

PSEQ was considered the Intercept for RQ4. The *p*-value < 0.001 on the PHQ-9 indicated the PHQ-9 was a statistically significant predictor of PSEQ. Post hoc analysis of this model with residual plots and variance inflation demonstrated no significant issues in model appropriateness. The association between PHQ-9 and PSEQ was statistically substantiated in the adjusted model. The next step required fitting the model to produce the association between PHQ-9 on PSEQ. Multiple regressions included both depression and pain self-efficacy to demonstrate the impact of both within the context of reported disability. The ODI acted as the Intercept in Table 6. The estimate of PHQ-9 was no longer considered significant when analysis demonstrated a *p*-value = 0.839. Additionally, the estimate effect of PSEQ demonstrated a *p*-value = < 0.001 when regressed against the ODI. This indicated full mediation by PSEQ on the relationship between depression and reported disability. Based on this analysis, a sufficient framework completed the mediation analysis with a model including PHQ-9 and PSEQ. This was demonstrated by finding that the depression no longer had a statistically significant relationship with a reported disability. A post hoc analysis was performed. The Q-Q plot of residuals indicated some minor non-normality though insufficient. The *p*-values were reliable. Likewise, the variance inflation factors were slightly higher on this model, but well under the standard acceptable limit. Finally, there were no residuals with leverage that would have substantially impacted these results.

**Table 6:** ODI, Pain, Age, PHQ-9 and PSEQ Model Coefficients

95% Confidence Interval							
Predictor	Estimate	***SE***	Lower	Upper	***t***	***p***	Stand. Estimate
Intercept	39.1147	7.5520	24.0993	54.130	5.179	< 0.001	0.3461
Pain Level	2.8073	0.5936	1.6271	3.988	4.729	< 0.001	
Age	0.1863	0.0857	0.0159	0.357	2.174	0.033	0.1266
PHQ-9	0.0327	0.1609	−0.2872	0.353	0.203	0.839	0.0154
PSEQ	−0.6610	0.0911	−0.8420	−0.480	−7.258	< 0.001	− 0.5871

The third research question determined that pain self-efficacy fully mediated the relationship between pain catastrophizing and reported disability when adjusting for pain level and age. This suggested that pain self-efficacy was the mechanism through which catastrophizing affects disability. The assumptions of this model were further validated through post hoc analysis of Q-Q plot residuals and variance inflation calculations. There was minor evidence of non-normality in the Q-Q plot, but not substantial enough to question the *p*-values on the results.

The fourth research question determined that pain self-efficacy fully mediated the relationship between depression and reported disability when adjusting for pain level and age. This suggested that pain self-efficacy is the mechanism through which reported levels of depression affects disability. The assumptions of this model were further validated through post hoc analysis of Q-Q plot residuals and variance inflation calculations. There was minor evidence of non-normality in a couple of Q-Q plots, but not substantial enough to question the *p*-values on the results.

## Discussion

**Fig. 2:**
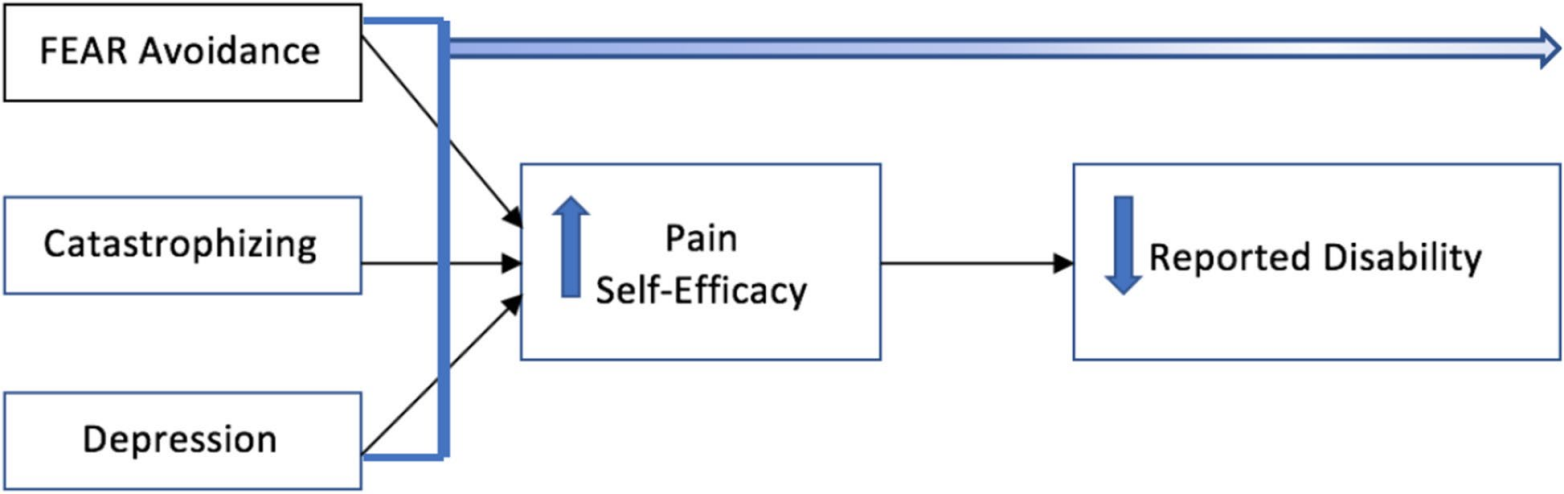
Model for pain self-efficacy’s inverse relationship with reported disability and its mediating relationship with fear, pain catastrophizing, and depression

The principal findings of the study were as follows. First, a strong inverse relationship between pain self-efficacy and reported disability was established. Further, pain self-efficacy was strongly considered to have a mediating role between reported fear of physical activity and disability, reported pain catastrophizing and disability, and reported depression and disability. Additionally, adjusting for age and reported pain levels proved to be statistically significant though it did not alter pain self-efficacy’s mediating role (Fig. 2).

Mediation refers to a causal model, whereas the mediator was the factor presumed to cause the outcome [39]. The following theoretical interpretations are premised on the empirical mediation analysis. The statistical significance of age and reported pain levels required statistical adjustments. The statistical suggestion for the combination of pain level and age adjustments reflected potential for greater levels of reported disability with lower levels of pain self-efficacy. The higher the pain level and the greater the age, the more significant the reported disability and the lower the pain self-efficacy. Regardless, the principal findings were not affected, partly due to the cut off age of 60. The findings supported the hypotheses that pain self-efficacy clearly influence and potentially mediate the relationships between specific psychosocial factors and reported disability to a greater degree than previously understood. The finding that pain self-efficacy has a mediating role between the three psychosocial factors and reported disability agree with multiple studies that individually investigated these relationships in musculoskeletal pain patients [15, 23, 35, 60–63].

This investigation of mediation analysis between specific psychosocial factors and pain self-efficacy was the first that could be identified. Lower levels of pain self-efficacy present with elevated levels of reported pain and disability at nearly a one-to-one ratio. Likewise, higher levels of pain self-efficacy are related to better functional outcomes [15, 35, 60, 64]. Pain self-efficacy accounts for the interdependent relationship between reported pain levels, psychological factors, and reported disability. Prior to this understanding, there was only moderate evidence supporting the predictive and influential role psychosocial factors have over reported disability [15, 65]. Self-efficacy contributes to the current understanding of associations between psychosocial factors, functional outcomes, and chronic pain. These results are consistent with previous cross-sectional studies that investigated pain self-efficacy and concluded partial mediation between reported pain levels, psychosocial factors, and reported disability in chronic pain patients [35, 61, 66].

Previous studies identified that pain self-efficacy has an inverse relationship with functional performance [35, 62, 67]. This study’s similar conclusion was not a complete surprise. The distinction between the two is possibly linked to specific coping strategies. The two most congruent pain management coping strategies are adaptive and non-adaptive strategies, also known as confrontational and avoidant strategies. This study facilitates the understanding of how pain self-efficacy reconciles the relationship between psychosocial factors and coping strategies. For example, those who used avoidant coping skills often have lower levels of self-efficacy and higher levels of reported disability. This is also seen in those with higher levels of pain. The severity of pain may reflect lacking pain self-efficacy strategies [68]. The study highlights the potential that lower pain self-efficacy compromises the management of fear, catastrophizing, and depression in the context of pain. The emphasis of this manuscript is seen with the conclusion that pain self-efficacy’s inverse relationship with reported disability also serves as a potential mediator for the investigated psychosocial factors of fear, catastrophizing, and depression. This clarification helps direct treatment paradigms that attempt to address specific psychosocial factors. Pain self-efficacy may serve to unify treatment paradigms addressing psychosocial factors that are currently thought to influence chronic pain and reported disability [62, 69].

Pain-related fear is the immediate alarm to a perceived threat characterized by a reactive impulse to escape [70, 71]. It seems logical that fear avoidance behavior as a predictor of elevated disability is also recognized for its critical role in the development of chronic pain. The fear-avoidance model has been at the forefront of the literature explaining the central mechanism of chronic back pain and long-term LBP problems [72]. Avoidance behavior has significant consequences that increase the risk of developing chronic pain. The consequences include reinforced focus on pain, inefficient coping strategies, aberrant movement patterns, and overall deconditioning [71]. The logical associations between pain-related fear and poor outcomes provides a theoretical predictive model that also reflects a research bias. The principal investigations of fear consistently relate to predictive ability rather than mediating potential. In fact, there are studies that found fear may not have as strong of a relationship as previously conceived. A cross-sectional analysis that focused on the role of self-efficacy on the relationship of fear and catastrophizing on reported disability found no relationship between fear and the other variables in fibromyalgia patients [63]. Further, an earlier study identified the exact opposite of the aforementioned assertions. The Rhudy and Meagher (2000) study showed that associations of fear, strong negative affect, and intense arousal can inhibit pain. The fear-avoidance model is broad and inconclusive to consistently identify the specific role over the prediction and mediation of reported disability. It may be important to identify the difference between fear and pain self-efficacy. Fear-related pain behavior inhibits the potential for resilience. Moreover, poor pain self-efficacy promotes a loss of resilience while increasing helplessness. This study identifies that pain self-efficacy is a mediator between fear and reported disability. Only one other cross-sectional analysis, completed as a part of their longitudinal investigation, identified “pain self-efficacy beliefs were a stronger mediator of the relationship between pain intensity and disability than fear-avoidance beliefs” [35]. Costa et al’s [35] longitudinal study concluded pain self-efficacy beliefs had a greater contribution to reported disability outcomes than does fear of movement, pain intensity, and related psychosocial factors.

Previous and emerging research identifies that pain self-efficacy is the only independent variable that serves as a significant predictor of avoidance behavior [60, 73]. This emerging research characterizes fear as an immediate and contextual response. The implication may be that the cognitive aspect of fear can be short lived and other psychosocial factors consistent in chronic pain, such as distress, anxiety, and catastrophizing, are being misinterpreted. Originally, the term pain catastrophizing was provided to identify a potential antecedent to fear avoidant behavior [63, 74]. The contingency is that lower reported pain self-efficacy enables prolonged avoidance strategies, which elevate the presence of catastrophizing and fear. The literature investigating non-specific low back pain frequently implicates two factors as potential mediators between reported pain levels and reported disability, pain self-efficacy and kinesiophobia, or the fear of physical activity [35, 74]. Most of the literature focuses on the fear-avoidance model as a predictor rather than a mediator of reported disability [38]. Though much of Woby’s research also identified pain related anxiety and catastrophizing as precursors to pain-related fear [38]. This research did not replicate the suggestion that fear served as a mediator for reported disability when adjusted for age and reported pain levels. Denison’s statistical analysis demonstrated that the variance in reported disability was explained by self-efficacy rather than fear avoidance. These arguments are now highlighted by the results of this study, which asserts that pain self-efficacy exists as a potential mediator between fear, catastrophizing, and depression, and reported disability [62].

Any relationship between pain self-efficacy and fear has the potential to be evaluated as a compromise in coping strategies. Maladaptive coping strategies in chronic pain are potentially heard through catastrophic verbalization. The literature demonstrates that catastrophizing predicts an increase in pain and a decrease in functional ability [72, 75, 76]. Catastrophizing consists of three sub-scales: rumination, magnification, and helplessness. The totality of the sub-scales is generally considered a cognitive process, though authors assert the sub-scales also have an affective presentation [66, 77–80]. Catastrophizing relates to distress and anxiety, which activate the pain neuromatrix and escalate pain, amplify distress, and increase reported disability [29, 81]. Additionally, catastrophizing is further defined by a negative evaluation of one’s own ability to cope with pain, also expressed as helplessness [66, 79]. Helplessness is considered a unique predictor of affective pain presentations [82]. Helplessness mirrors poor pain self-efficacy through an individual’s consistent perception of an inability to control pain [83, 84]. While catastrophizing appears to be a cognitive process, the supportive evidence identifies helplessness as an affective disorder. In other words, catastrophizing could be both situational and dispositional. The disposition is a poor sense of control that is expressed through feelings of powerlessness to face a painful situation. Helplessness as an affective disorder is also supported by the variance that separates it from rumination and magnification [83, 85, 86]. However, an isolated correlation between helplessness and pain self-efficacy was not found in this manuscript. The manuscript identified that pain self-efficacy mediates the relationship between the three different catastrophizing subscales. In this regard, the relationship between catastrophizing and pain self-efficacy makes a good predictor of pain tolerance during functional activities. Therefore, pain self-efficacy helps facilitate an improved sense of personal control. The influence self-efficacy has over catastrophizing helps to prioritize the affective presentation and the associated cognitive distortions, facilitating a specific rehabilitation process for those enduring elevated levels of reported pain.

The theoretical, academic, and clinical challenge is to better understand the antecedent of situational and dispositional expressions of catastrophizing. For example, the relationship of catastrophizing to anxiety and fear makes it a consistent predictor of pain intensity and reported disability [72, 76, 87, 88]. The antecedent in these presentations is a situational stressor, specifically pain or the anticipation of pain that reflects a cognitive process. The attentional focus on pain in the presence of anxiety may lead to fear of pain. Further, the attentional focus on pain and the development of fear give rise to the underlying sense of helplessness [83]. The dispositional sense of helplessness presented in catastrophizing affirms pain self-efficacy and the potential development of depression [37, 83, 84]. This underlying sense of helplessness and poor self-efficacy are the affective dispositions that allow anxiety, catastrophizing, and fear to reach threshold levels that hijack one’s ability to actively cope with pain. Therefore, catastrophizing is conceptualized as both a cognitive and affective distortion in the presence of pain-related stressors including fear and depression [37].

This research asserts that pain self-efficacy is a potential mediator between catastrophizing and reported disability. A compromise in pain self-efficacy may serve as a point of reference that simultaneously limits coping possibilities and elevates catastrophizing. The scope of usable coping strategies becomes narrow in the presence of poor self-efficacy. Likewise, the exaggerated expression of pain and disability appear as the primary response, particularly with developing psychosocial factors such as anxiety, fear, and depression. Interestingly, a focus on cognitive restructuring of catastrophizing is required as one intervention to facilitate change in the level of pain self-efficacy [83, 89]. Modifying catastrophic thinking alters the point of reference. It makes sense that pain self-efficacy directly affects the perceptions of one’s ability to control pain [69]. A modification of catastrophic expressions then influences beliefs regarding ability and control.

Depression is the most common psychosocial factor of chronic pain. A unifying neurobiological theory is that depression and pain symptoms shared the same neural pathways and neurochemicals. The modulation of pain in the descending neuropathways are influenced by psychological mechanisms related to anxiety, depression, expectations, and attention [48, 81, 90]. Symptoms overlap between clinical depression and features in chronic pain conditions. Many of the central nervous system’s structures that process affective components of pain are the same pathways for depression [91]. Patients with depression and chronic low back pain often show higher levels of cognitive distortion than non-depressed patients with chronic low back pain. This cognitive distortion is associated with distress, anxiety, and disability. In this regard, depression has a strong influential role in chronic pain. Cognitive distortion is related to depression and disability and linked to CLBP. Therefore, the overlapping expressions of fear of physical activity, pain catastrophizing, and depression share overlapping similarities in chronic pain. Banks and Kerns [92] proposed a three-pronged model to help explain the pain-depression relationship: (a) pain-related interference, (b) cognitive-affective distortion, and (c) helplessness. This three-pronged model articulates the influence self-efficacy has over pain coping strategies [92]. The interpretation of an event such as pain that cannot be controlled through personal action is perpetuated through cognitive-affective distortions and contributes to the sense of helplessness. Sustained helplessness potentially leads to depression. Pain self-efficacy is theoretically a mediator between depression and compromise in function [93, 94]. The findings in this study are consistent with this assertion.

Pain self-efficacy and depression are categorized as affective traits. Affective traits and disturbances are associated with changes in behavior that place a person at risk for progression of pain, symptom severity, and reported disability. Increasing frustration and subsequent depression associated with perpetuating pain results in personal interference enabling disengagement from individual purpose and goals. This personal disengagement further compromises functional ability in the presence of poor pain self-efficacy. This manuscript suggests that pain self-efficacy potentially mediates the relationship between depression and reported disability. There is no determination of depression with the completion of the PSEQ. However, it could be declared that someone with CNLBP with depression would present with low pain self-efficacy. Therefore, addressing pain self-efficacy influences the level of depression. Previous studies identified that improvements in self-efficacy are associated with improvements in depression [95]. This is specifically identified by Skidmore et al’s [15] study that found changes in pain self-efficacy explained changes in depression as well as changes in the affective interpretation of pain. Increased confidence in the ability to accept pain, tolerate activity, and improve function for daily activities despite elevated pain reports relates to a decrease in depression [15]. The importance can now be placed on targeting pain self-efficacy and underlying confidence in personal beliefs in one’s own abilities to cope with pain [61]. The same cannot be accomplished by treating depression in isolation. Previous research shows that the contribution of pain self-efficacy, greater than reported pain levels and disability alone, had the most weight when determining how or why someone becomes depressed. In this regard, pain self-efficacy takes priority as it holds potential mediating affect over depression and reported disability. It remains critical to point out that the findings imply treatment targeting depression is required to fully affect reported disability.

### Limitations

Certain limitations associated with this study must be acknowledged. The identified literature that helped develop the construct and methods for this research used a diverse amount of outcome measures. There are multiple examples of research that used different self-reported questionnaires to measure similar outcomes. The opportunity to establish meaningful comparisons between studies became limited in the presence of multiple questionnaires investigating the same variable. Further, the investigation of pain self-efficacy is nearly as varied as self-reported measures though the underlying theoretical construct of self-efficacy is relatively consistent. For example, some articles referred to functional self-efficacy while others referred to coping self-efficacy. Likewise, the division of the subscales within the Fear Avoidance Behavior Questionnaire offers a similar limitation. The FABQpa reflects a single construct of fear avoidance behavior models that are built on multiple constructs. A wider range of fear avoidance constructs may provide improved observations. Additionally, the instruments used for this study required subjective reporting. The instruments used are valid and reliable, though they still require an assumption that those completing them are forthright. However, reported bias cannot be avoided. The delimitations of this research reflect how the research did not investigate the totality of psychosocial factors. For example, distress, anxiety, anger, and coping are not specifically included in the investigation, though they are included in part of the discussion. It is possible that the overlapping meaning between all the psychosocial factors are misrepresented by the participants than otherwise intended. It is also important to point out that psychosocial factors have a fluid nature of influence, whereas they have varying psychological and behavioral impacts at different points in a person’s life. Participants were also recruited through multiple hospital-based clinics, private practices, and primary care facilities that are providing or promoting rehabilitation services to individuals with chronic pain, which potentially impacts psychological and behavioral interpretations. The sample is at risk of being a non-probability sample, thus inferences regarding the generalizations of findings to different populations are made with extreme caution. Finally, mediation analysis is best carried out with the potential for directional preference through longitudinal analyses. Mediation analysis is primarily utilized to identify causal mechanisms, in order to avoid any inflation of the correlations. This is not completely possible through the cross-sectional design of this study, though the evolution of statistics allowed for theoretical assertions. The results of this research should be taken with thoughtfulness, as an assumption of the directional pathways of causality can be determined. Nevertheless, none of the studies found and reviewed specifically evaluated the possible mediation effect of pain self-efficacy between several psychosocial factors and reported disability in non-specific low back pain patients.

### Implications for practice

This research offers a reasonable expectation that every patient with CNLBP could be offered the PSEQ. The clinical interpretation of the questionnaires predicts levels of resilience or risk. This needs to be clearly understood to target the interventions. Performance levels related to a person’s self-efficacy helps determine the plan of care and the outcome. The clinical implication is summarized as a critical need to target pain self-efficacy within a pain management context. Targeting pain self-efficacy is reinforced with problem-solving skills, goal setting, and performance mastery. Further, self-efficacy through goal setting and graded accomplishment correlate with predictions of reported disability [96]. The experience of performance accomplishment is created by encouraging patients to commit to sub-tasks that are graded toward higher levels of complexity and reflect a series of personal goals. These tasks enable confidence in task management in the presence of pain. In this case, the promotion is toward coping behaviors that are mediated by a person’s beliefs and confidence in their ability. Self-efficacy is viewed as a contextual concept that is likely to vary with personal experiences and dynamic interactions. As clinicians target pain self-efficacy in pain management, they need to challenge themselves to creatively think of ways to encourage confidence building.

### Recommendations for future research

The principal recommendation for future research is a longitudinal analysis, which takes the conclusion beyond theoretical assertions. A study that prioritizes a longitudinal investigation of pain self-efficacy’s mediating role could include additional psychosocial factors such as distress, anxiety, and anger. Clinical implications for developing and improving pain self-efficacy could be enhanced by additional longitudinal studies that investigates problem-solving skills, goal setting, and graded task accomplishment for individuals with chronic pain and high disability. Skill acquisition regarding independent pain management that involves problem-solving skills and goal setting would begin to capture the true mediation effect of pain self-efficacy. Such a research agenda could conclude the totality of the significance of pain self-efficacy in the rehab setting.

## Conclusion

Overall, the results of this study indicated that pain self-efficacy is an important factor in the relationship between psychosocial factors of fear, catastrophising, and depression and pain-related disability. The assertion is that adaptive coping strategies premised on personal confidence is promising for those managing chronic pain. The implication is that every patient in chronic pain that enters a physical therapy outpatient facility must be screened for pain self-efficacy. Knowledge concerning the relative importance of each of these screening tools and overlapping factors is necessary for understanding effective psychosocial evaluations, which allow insight into the influences on emotional overlay, perceived function, and expected outcomes. An opportunity for clinicians to implement this research includes teaching patients to build a repertoire of skills to improve their overall confidence, including self-efficacy to independently manage pain and optimally function in daily life.

## Data Availability

The protocols for the research and associated data set used and/or analysed during this study are available from corresponding author on reasonable request. The datasets generated during and/or analysed during the current study are available in Clinical Trial Sharing Website repository.

## Abbreviations

CLBP: Chronic low back pain
CNLBP: Chronic non-specific low back pain
FABQ: Fear Avoidance belief Questionnaire
FABQpa: Fear Avoidance Belief Questionnaire physical activity
FABQw: Fear Avoidance Belief Questionnaire work
ODI: Oswestry Disability Index
PCS: Pain Catastrophizing Scale
PHQ-9: Patient Health Questionnaire – 9
PSEQ: Pain Self-Efficacy Questionnaire
RQ1: Research Question 1
RQ2: Research Question 2
RQ3: Research Question 3
RQ4: Research Question 4
